# Obesity Hypoventilation Syndrome in Children and Adolescents

**DOI:** 10.3390/children13010140

**Published:** 2026-01-18

**Authors:** Duilio Petrongari, Paola Di Filippo, Francesca Cacciatore, Armando Di Ludovico, Giuseppe Francesco Sferrazza Papa, Sabrina Di Pillo, Francesco Chiarelli, Marina Attanasi

**Affiliations:** 1 Pediatric Allergy and Pulmonology Unit, Department of Pediatrics, University of Chieti-Pescara, Via dei Vestini n°5, 66100 Chieti, Italy; duilio.petrongari@studenti.unich.it (D.P.); francesca.cacciatore@studenti.unich.it (F.C.); armando.diludovico@studenti.unich.it (A.D.L.); sabrina.dipillo@asl2abruzzo.it (S.D.P.); chiarelli@unich.it (F.C.); marina.attanasi@unich.it (M.A.); 2 Department of Neurorehabilitation Sciences, Casa di Cura Igea, 20144 Milan, Italy; giuseppe.sferrazza@unikore.it

**Keywords:** obesity hypoventilation syndrome, pediatric obesity, sleep-disordered breathing, obstructive sleep apnea syndrome

## Abstract

Obesity hypoventilation syndrome (OHS) is a severe and underrecognized respiratory disorder characterized by the coexistence of obesity, daytime hypercapnia, and sleep-disordered breathing. Although well described in adults, pediatric OHS remains poorly defined despite the rising prevalence of childhood obesity. Its pathophysiology is multifactorial, involving obesity-related mechanical constraints, impaired ventilatory control, altered chemosensitivity, and frequent overlap with obstructive sleep apnea. Clinical manifestations in children are often subtle and nonspecific, including snoring, sleep fragmentation, daytime sleepiness, and neurocognitive impairment, frequently leading to delayed diagnosis and, in some cases, acute cardiopulmonary decompensation. Management of pediatric OHS is challenging and largely extrapolated from adult data. Positive airway pressure therapy remains the cornerstone of treatment, while weight reduction is essential but difficult to achieve in pediatric populations. Pharmacological approaches such as medroxyprogesterone or acetazolamide remain experimental, with limited pediatric evidence. This review synthesizes current knowledge on pediatric OHS, focusing on epidemiology, pathophysiology, clinical presentation, diagnostic challenges, and therapeutic strategies. Increased awareness and earlier recognition are essential to prevent progression to chronic respiratory failure and long-term cardiovascular complications.

## 1. Introduction

The prevalence of childhood obesity has increased dramatically over recent decades, reaching epidemic proportions worldwide. According to data from the U.S. Centers for Disease Control and Prevention (2017–2020), obesity affects 12.7% of children aged 2–5 years and 20.7% of those aged 6–11 years. Excess adiposity in childhood is strongly associated with respiratory morbidity, including obstructive sleep apnea syndrome (OSAS) [1,2], asthma, and obesity hypoventilation syndrome (OHS) [3], and contributes to long-term cardiometabolic risk [4].

In addition to obesity, early-life factors may further increase vulnerability to respiratory disease. Children born extremely or very preterm often exhibit persistent impairments in lung structure and gas exchange during school age, which may predispose them to obesity-related ventilatory disorders later in life [5].

Although OHS has been extensively described in adults, its occurrence in children and adolescents remains poorly defined. The available pediatric data suggest that OHS may be underrecognized rather than truly rare. In a French retrospective cohort of 102 obese children (mean age 10.5 years), Gachelin et al. [4] reported symptoms suggestive of OSAS in nearly one-third of participants, while OHS was diagnosed in 3.9% of the cohort. Notably, OHS frequently coexisted with OSAS, highlighting the diagnostic overlap between these conditions.

OHS is classically defined in adults by the triad of obesity, sleep-disordered breathing, and chronic daytime hypercapnia (PaCO_2_ ≥ 45 mmHg at sea level), in the absence of alternative causes of hypoventilation [6]. In pediatric populations, obesity is defined using age- and sex-specific criteria, typically as a body mass index (BMI) at or above the 95th percentile [7,8].

Given the increasing burden of pediatric obesity and the potential severity of OHS (ranging from neurocognitive impairment to cardiopulmonary complications) greater awareness and earlier recognition are urgently needed. This narrative review provides an updated overview of OHS in children and adolescents, focusing on epidemiology, pathophysiology, clinical presentation, diagnostic challenges, and management strategies. By integrating the available pediatric evidence with adult and experimental data, we aim to highlight disease-specific features, identify key unresolved issues, and outline priorities for future research. Increased awareness and earlier recognition are essential to prevent progression to chronic respiratory failure and long-term cardiopulmonary complications in this vulnerable population.

## 2. Materials and Methods

A comprehensive narrative literature review was conducted to identify studies relevant to OHS in children and adolescents. The search was performed using PubMed/MEDLINE and included publications from January 1995 to October 2025. Given the narrative scope of the review, other databases were not systematically searched.

The primary search strategy used combinations of the following terms: “obesity hypoventilation syndrome” OR “OHS” AND “children”, “adolescents”, or “pediatric”; “pediatric obesity” AND “hypoventilation”; “sleep-disordered breathing” OR “pediatric OSAS” AND “hypercapnia”; “pediatric ventilatory control”; and “leptin resistance” AND “obesity”.

Additional topic-specific keywords were applied for individual sections of the review. These included terms related to lung mechanics and respiratory muscle function in pediatric obesity; non-invasive ventilation (“pediatric CPAP”, “pediatric BiPAP”, “home mechanical ventilation”); weight-management strategies (“lifestyle intervention”, “bariatric surgery”); and pharmacological approaches (“acetazolamide”, “medroxyprogesterone”) in pediatric hypoventilation.

Eligible publications included narrative and systematic reviews, observational studies (prospective and retrospective cohorts), randomized and non-randomized clinical trials, case series, and clinically relevant case reports involving individuals aged 0–18 years. Studies focused exclusively on adult populations without clear relevance to pediatric disease were excluded, as were reports of acute hypercapnic respiratory failure unrelated to obesity. Only articles published in English were included, with the exception of one relevant retrospective pediatric study published in French.

Study selection was based on relevance to pediatric OHS pathophysiology, diagnosis, and management. Titles and abstracts were screened initially, followed by full-text review when appropriate. Reference lists of included articles were also manually searched to identify additional relevant publications.

As this was a narrative review, no formal methodological quality assessment or quantitative data synthesis (e.g., meta-analysis) was performed. The pediatric evidence base was limited and highly heterogeneous, consisting predominantly of small retrospective cohorts, case series, and isolated case reports. This limitation was taken into account when interpreting the findings.

The initial search identified approximately 1598 records. After title and abstract screening, 95 articles underwent full-text assessment, and 20 publications were ultimately included. Across these studies, the total number of reported pediatric patients with OHS was small and fragmented, precluding reliable aggregation of patient-level data or precise estimation of the overall pediatric disease burden.

## 3. Epidemiology and Risk Factors

The true prevalence of OHS remains uncertain, even in adults. In adult populations, prevalence estimates range from approximately 0.3–0.4% in the general population and increase to 8–20% among adults with obesity [9,10]. No population-based prevalence estimates are currently available for children and adolescents, and the true burden of pediatric OHS remains unknown. Most available information comes from small, retrospective cohorts of obese children evaluated for sleep-disordered breathing, in which OHS is likely underrecognized due to nonspecific symptoms and the lack of validated pediatric diagnostic criteria [11,12].

The rising prevalence of pediatric obesity represents a major public health concern and is strongly associated with long-term respiratory morbidity [13], which may predispose children to OHS, particularly when other factors affecting lung function, such as prematurity, are present [14]. Although ethnic variations in OHS have been suggested, no ethnicity-specific risk factors have been definitively established [15]. Notably, the higher prevalence of morbid obesity among African Americans may contribute to their increased susceptibility to OHS, as suggested by epidemiological data on obesity rates [16] and clinical observations in OHS populations [15].

Hu et al. [17], in a cross-sectional study of 1467 children, observed higher rates of obesity among African American and Hispanic children aged 2–5 years compared with non-Hispanic, non-African American peers (12.1% and 21.1% vs. 10.8%, respectively). These demographic disparities may indirectly influence OHS risk in childhood through obesity-associated mechanical and metabolic effects on ventilation.

Geographic differences also appear relevant. Harada et al. [18], studying 981 adults with suspected OSAS in Japan, reported that BMI among Japanese patients with OHS was lower than that typically observed in Western cohorts, suggesting that body fat distribution and ethnicity may modulate the development of obesity-related ventilatory impairment. These observations should be regarded as hypothesis-generating, as ethnicity- and geography-related differences in OHS have not been specifically confirmed in pediatric populations.

Several risk and protective factors for pediatric OSAS have been identified across the developmental spectrum, including obesity, craniofacial abnormalities, neuromuscular tone, and early-life influences, all of which contribute to substantial interindividual variability in disease expression [2]. Sex-related patterns differ between OHS and OSAS: while OHS appears to affect males and females more equally in clinical cohorts [19], OSAS shows a clear male predominance [2]. These differences underscore that, although obesity remains the primary risk factor for OHS, its epidemiology is shaped by demographic and anthropometric determinants that differ from those implicated in OSAS alone.

Given the scarcity of pediatric data, further research is urgently needed to clarify the prevalence, clinical spectrum, and determinants of OHS in children and adolescents. Early identification of obesity-related and demographic risk factors may facilitate timely diagnosis in high-risk pediatric populations, improve clinical outcomes, and prevent progression to chronic respiratory failure.

Available pediatric cohorts reporting obesity hypoventilation syndrome are reported in Table 1.

**Table 1:** Available pediatric cohorts reporting obesity hypoventilation syndrome. No population-based studies specifically designed to estimate the prevalence of pediatric OHS have been published to date. Most available data derive from selected cohorts. Adult prevalence estimates (0.3–0.4% in the general population and 8–20% among individuals with obesity) are reported for contextual comparison only and do not reflect pediatric prevalence, which remains unknown. This table is intended as a descriptive summary; heterogeneity and small sample sizes limit direct comparability.

Study	Country	Study Design	Population	Sample Size	Diagnostic Criteria	OHS Prevalence
Gachelin et al. [4]	France	Retrospective cohort	Obese children referred for sleep evaluation	102	Adult OHS criteria applied (BMI, OSAS, PaCO_2_ ≥ 45 mmHg)	3.9%
Witmans et al. [7]	Canada	Case series	Obese children with sleep-disordered breathing	Small cohort	Adult criteria extrapolated	Not population-based
Other reports	Various	Case reports/small series	Severely obese children	<10 each	Heterogeneous	Not estimable

## 4. Pathophysiology of Obesity Hypoventilation Syndrome

**Figure 1:**
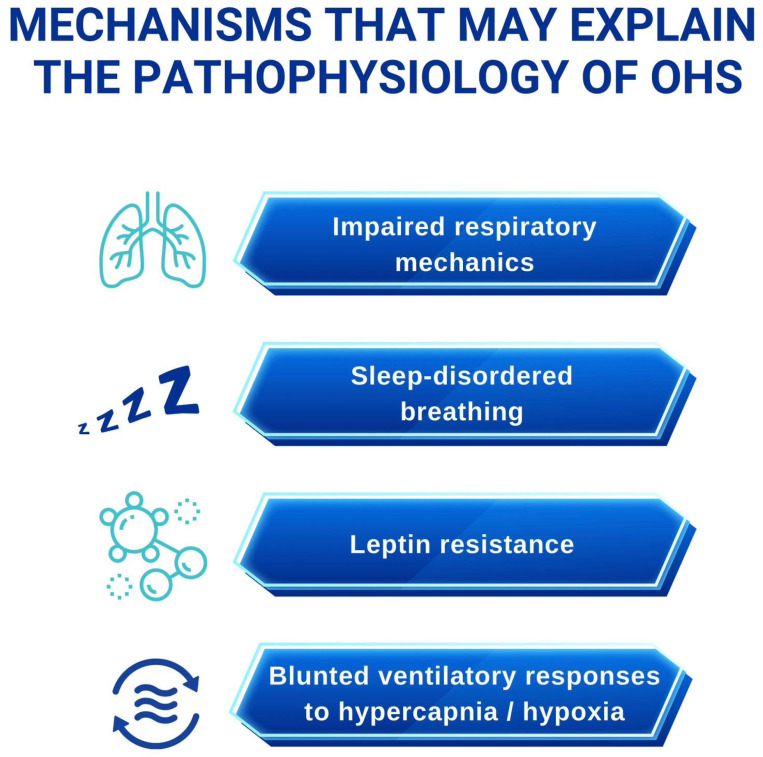
Mechanisms underlying the pathophysiology of Obesity Hypoventilation Syndrome in children.

The pathogenesis of OHS is multifactorial and reflects the interplay between obesity-related mechanical constraints, impaired ventilatory control, abnormalities in gas exchange, and neurohormonal dysregulation [9]. These factors converge to produce chronic alveolar hypoventilation, most evident during sleep but progressively extending into wakefulness. Mechanisms underlying the pathophysiology of OHS in children are summarized in Figure 1.

### 4.1. Obesity-Related Mechanical Load and Pulmonary Function Impairment

Excess adipose tissue in the thoracic and abdominal regions profoundly alters respiratory mechanics by reducing lung, chest wall, and overall respiratory system compliance [16,17]. In obese individuals, respiratory system compliance is reduced by approximately 20% compared with eucapnic obese subjects and by up to 60% in those with OHS relative to normal-weight individuals [20]. Fat accumulation around the rib cage and abdomen increases intrapleural pressure, restricts diaphragmatic excursion, and limits chest wall expansion, leading to marked reductions in expiratory reserve volume (ERV), functional residual capacity (FRC), and tidal volume [21].

While eucapnic obese individuals typically compensate for these mechanical constraints by increasing respiratory rate and neural respiratory drive, patients with OHS exhibit an attenuated ventilatory response to hypoxia and hypercapnia, resulting in inadequate minute ventilation and progressive CO_2_ retention [20,22]. Increased upper-airway resistance further amplifies the mechanical burden. Hypercapnic patients demonstrate elevated airway resistance in both sitting and supine positions, contributing to increased work of breathing [23,24]. In obesity, the supine position further reduces FRC, promotes expiratory flow limitation, and worsens ventilation–perfusion mismatch, facilitating small-airway closure and intrinsic positive end-expiratory pressure [21,25].

As a consequence, the inspiratory threshold load increases and respiratory muscle efficiency declines. Work of breathing is significantly higher in patients with OHS than in normal-weight individuals or eucapnic obese patients with OSAS [22,23]. Chronic hypercapnia, hypoxemia, and persistent diaphragmatic overload may adversely affect respiratory muscle function, particularly in children whose respiratory systems are still developing [24,26]. Collectively, reduced lung volumes, increased airway resistance, and impaired respiratory muscle efficiency create a mechanical environment that predisposes obese children to alveolar hypoventilation and chronic ventilatory failure [26].

Pediatric implications: In children and adolescents, obesity-related mechanical constraints may have a greater functional impact due to ongoing lung growth and chest wall development. However, most quantitative data on respiratory system compliance and work of breathing in OHS derive from adult studies, and pediatric-specific physiological measurements remain limited.

### 4.2. Interaction Between OHS and Obstructive Sleep Apnea

OSAS is present in approximately 90% of adults with OHS [15] and affects 1–5% of children [27], with obesity representing a major risk factor in the pediatric population. Overweight and obese children have a 24–61% higher risk of OSAS than their normal-weight peers [28]. This is largely due to adipose deposition in peripharyngeal tissues, which promotes dynamic upper airway collapse during sleep, and obesity-related reductions in functional residual capacity that further predispose to obstruction [2]. Anthropometric markers such as neck-to-waist ratio [29] and BMI-related measures, particularly during adolescence [30], may help identify individuals at increased risk for OSAS and, consequently, for OHS.

Recurrent obstructive events lead to chronic hypercapnia through repeated cycles of apnea-induced CO_2_ retention, brief arousals, and incomplete ventilatory compensation [31]. Sleep fragmentation and chronic intermittent hypoxia progressively attenuate central respiratory drive, reducing ventilatory responsiveness to both hypoxia and hypercapnia [31,32]. Experimental studies by Ayappa et al. [33] and Berger et al. [34] demonstrated that chronic hypercapnia is associated with a diminished post-event ventilatory response, particularly when apneas are prolonged relative to inter-apnea intervals. Over time, this impaired compensatory mechanism results in morning hypercapnia that may persist throughout the daytime [35].

Although obesity alone increases the work of breathing and normally elicits a compensatory increase in neural respiratory drive to maintain eucapnia [36,37], this response is blunted in OHS, where both hypoxic and hypercapnic ventilatory responses are reduced [22]. The heightened respiratory drive in uncomplicated obesity is reversible with weight loss, supporting its adaptive nature [38]. In contrast, the reduced chemosensitivity in OHS appears to be acquired rather than inherited, as first-degree relatives of affected patients do not show abnormalities in ventilatory chemoreactivity [39].

Renal bicarbonate retention, as a compensatory response to chronic CO_2_ retention, may further suppress ventilatory drive [35]. Simulation studies by Norman et al. [40] demonstrated that impaired CO_2_ responsiveness combined with slower renal bicarbonate excretion facilitates progression from nocturnal to chronic daytime hypercapnia, highlighting a synergistic interaction between altered central respiratory control, renal adaptation, and obstructive sleep-disordered breathing. In children, whose ventilatory control systems are still maturing, OSAS-related hypoxia and sleep disruption may have an even greater impact on respiratory drive, potentially accelerating the transition from OSAS to OHS.

Pediatric implications: In pediatric populations, the high prevalence of OSAS in obese children suggests that sleep-disordered breathing may play a particularly important role in the progression toward OHS. Nevertheless, the mechanisms linking chronic intermittent hypoxia, impaired ventilatory drive, and the transition from OSAS to OHS in children are largely extrapolated from adult and experimental data.

### 4.3. Role of Hypoxemia and Neurotransmitter Imbalance

Nocturnal and chronic hypoxemia may modify neurotransmitter systems involved in ventilatory control. Experimental models have demonstrated alterations in adenosinergic [41] and GABAergic signaling [42] in obesity-related hypoventilation, providing a possible mechanistic link between hypoxia, altered chemosensitivity, and reduced respiratory drive. Prolonged stress and impaired sleep quality could further destabilize neurorespiratory control and intensify nocturnal hypoventilation [43], potentially accelerating the transition from OSAS to OHS.

Pediatric implications: Evidence linking hypoxemia-induced alterations in adenosinergic and GABAergic signaling to hypoventilation is derived primarily from experimental and adult studies. Direct confirmation of these neurochemical mechanisms in children with OHS is currently lacking.

### 4.4. Leptin Resistance and Neurohormonal Dysregulation

Leptin, an adipocyte-derived hormone, plays a central role in energy homeostasis and ventilatory regulation by stimulating central respiratory drive, enhancing upper-airway patency, and increasing metabolic rate [44]. Studies in leptin-deficient animal models have shown that leptin supplementation improves ventilation, increases CO_2_ sensitivity, and mitigates respiratory muscle abnormalities, highlighting its importance in respiratory control [45,46].

In humans, severe obesity is typically associated with hyperleptinemia, reflecting central leptin resistance rather than deficiency. Elevated leptin levels have paradoxically been linked to reduced ventilatory drive and impaired hypercapnic responsiveness, suggesting that leptin resistance contributes to blunted chemosensitivity and diminished ventilatory adaptation to increased mechanical load [47,48].

Leptin resistance may also exacerbate obesity-related reductions in lung volumes and respiratory system compliance, further increasing mechanical constraints on ventilation [20,49]. Although pediatric-specific data are limited, neurohormonal dysregulation likely plays a contributory role in the development of OHS in children, particularly in the context of severe obesity and coexisting sleep-disordered breathing.

Pediatric implications: Although leptin resistance is well documented in pediatric obesity, its specific contribution to impaired ventilatory control and OHS development in children has not been systematically studied. Current understanding of leptin-mediated respiratory dysfunction in pediatric OHS is therefore largely inferred from adult and animal models.

## 5. Clinical Presentation

OHS presents with a heterogeneous spectrum of respiratory and systemic manifestations, occurring during both sleep and wakefulness [50]. In children, symptoms are often subtle, nonspecific, and easily misattributed to obesity or behavioral issues, contributing to delayed recognition [7].

Nocturnal manifestations commonly include persistent snoring, episodes of obstructive or mixed apnea, nocturnal hypoventilation with consequent hypercapnia, and intermittent oxygen desaturations. These events frequently trigger repeated arousals, leading to fragmented sleep architecture. Parents may report night sweats, restless sleep, or observed apneas [51].

Daytime symptoms reflect the cumulative impact of chronic sleep disruption and impaired gas exchange. Affected children often experience excessive daytime sleepiness, morning headaches, chronic fatigue, reduced attention, and impaired concentration [7].

Importantly, the clinical presentation of pediatric OHS overlaps extensively with that of OSAS. Most nocturnal manifestations (including habitual snoring, witnessed apneas, sleep fragmentation, and intermittent oxygen desaturations) are indistinguishable from those observed in children with OSAS alone. Similarly, daytime symptoms such as excessive sleepiness, morning headaches, fatigue, and neurobehavioral disturbances are common to both conditions and are frequently attributed to uncomplicated OSAS or obesity itself [11,12]. This substantial overlap contributes to the underrecognition of OHS, particularly when daytime gas exchange is not systematically assessed. Persistent daytime hypercapnia and evidence of chronic hypoventilation therefore represent the key clinical features distinguishing OHS from isolated OSAS in obese children. Neurocognitive and behavioral consequences, such as irritability, poor academic performance, and attention deficits, may mimic other developmental or psychiatric conditions, increasing the risk of misdiagnosis.

In more advanced or prolonged diseases, children may develop clinical signs of chronic hypoxemia, including cyanosis, digital clubbing, and exercise intolerance. Peripheral edema and signs of right-sided heart failure can occur, reflecting the progression to pulmonary hypertension [52].

Because these manifestations overlap with those of OSAS, asthma, and other common pediatric conditions, the diagnosis of OHS is frequently delayed. In some cases, the disorder is recognized only during acute episodes of respiratory or even cardiac decompensation, underscoring the need for heightened clinical vigilance [12]. Early identification is essential to prevent progression to chronic respiratory failure and cardiometabolic complications. Clinical and pathophysiological differences between OHS and OSAS are shown in Table 2.

## 6. Diagnosis of Obesity Hypoventilation Syndrome in Children and Adolescents

Pediatric OHS results from mechanical constraints, impaired ventilatory drive, sleep-disordered breathing, and neurohormonal dysregulation. Children may develop chronic hypercapnia faster than adults due to less effective compensatory mechanisms, including renal bicarbonate handling and ventilatory plasticity. Early identification of at-risk children based on anthropometry, sleep-disordered breathing, and metabolic profiles is crucial to prevent respiratory failure.

Diagnosing OHS in children requires a stepwise, practical approach. Arterial blood gas analysis, while the gold standard, may not always be feasible as a first-line test in pediatric practice.

OHS is a diagnosis of exclusion, defined by obesity, sleep-disordered breathing, and chronic daytime hypercapnia, with other causes of hypoventilation ruled out [6,9]. Diagnosing children is particularly challenging because age-specific criteria are lacking, and overlap with obesity-related respiratory disorders, especially OSAS, is common [7].

In children, obesity is defined as BMI ≥ 95th percentile for age and sex, rather than the adult BMI threshold [53].

Daytime hypercapnia should be confirmed by arterial or capillary blood gas, showing PaCO_2_ ≥ 45 mmHg during wakefulness in stable conditions [15,22]. Elevated serum bicarbonate alone is not sufficient for diagnosis but supports chronic CO_2_ retention due to renal compensation [35].

Other causes of alveolar hypoventilation must be ruled out, including neuromuscular disease, chest wall disorders, central hypoventilation syndromes, and significant pulmonary disease (severe asthma, cystic fibrosis, interstitial lung disease) [9,31]. Careful history, neurological exam, and targeted investigations, including pulmonary function tests and, when indicated, neuroimaging or genetic testing, are required [7].

Polysomnography with integrated CO_2_ monitoring is key for documenting sleep-related hypoventilation, obstructive events, and nocturnal hypercapnia [27]. Continuous or transcutaneous CO_2_ monitoring is strongly recommended, as oxygen desaturation alone may underestimate hypoventilation severity [9]. In suspected pediatric OHS, polysomnography often shows prolonged nocturnal hypercapnia with obstructive or mixed apneas and inadequate post-event ventilatory compensation [31,34]. Persistent daytime hypercapnia distinguishes OHS from pediatric OSAS, where PaCO_2_ is usually normal [22].

Early recognition is critical because immature ventilatory control in children can lead to rapid progression to chronic respiratory failure, pulmonary hypertension, and cardiovascular complications [58]. Echocardiography may be used at baseline and follow-up to screen and monitor pulmonary hypertension. A high index of suspicion is warranted in severely obese children with OSAS, unexplained daytime sleepiness, morning headaches, or elevated serum bicarbonate, even without overt respiratory symptoms [7].

## 7. Treatment

Unlike OSAS, primarily managed by addressing upper-airway obstruction [59], pediatric OHS requires a broader approach targeting chronic alveolar hypoventilation, typically via non-invasive ventilation and comprehensive weight management. The management of OHS is complex, multidisciplinary, and individualized, aiming to normalize gas exchange and prevent pulmonary hypertension or chronic respiratory failure [4]. High-quality pediatric-specific evidence is limited, with most recommendations extrapolated from adult studies, small case series, or expert opinion [7]. This limitation underscores the need for careful adaptation of adult-derived protocols when applied to growing children, taking into account developmental physiology and the potential for long-term consequences.

Furthermore, pediatric OHS management presents unique challenges beyond those observed in adults. Adherence to positive airway pressure (PAP) therapy is often limited in children and adolescents due to discomfort, mask fit issues, and lifestyle constraints. In a retrospective cohort study, up to 25% of adolescents remained hypercapnic despite nightly PAP use [60]. These figures may not generalize to all centers. Psychosocial factors, including body-image concerns, low motivation, and family dynamics, can hinder engagement with lifestyle interventions [60]. Access to bariatric surgery is highly restricted by age, comorbidities, and psychosocial readiness, limiting its utility as a widespread intervention [61]. These factors highlight the need for individualized, multidisciplinary approaches combining respiratory support, family-centered weight management, and psychosocial support to optimize outcomes.

**Figure 2:**
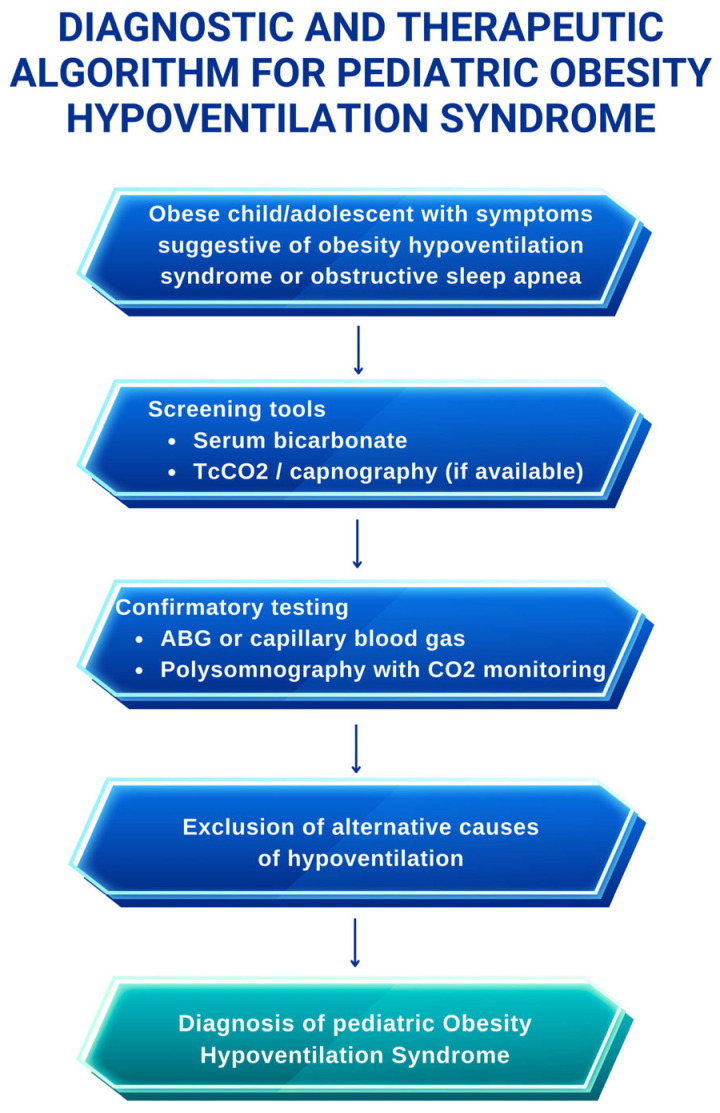
Diagnostic and therapeutic algorithm for obesity hypoventilation syndrome in children.

A diagnostic and therapeutic algorithm for OHS in children is shown in Figure 2.

### 7.1. Positive Airway Pressure Ventilation

PAP therapy represents the standard of care in pediatric OHS; however, optimal settings and adherence strategies are largely extrapolated from adult protocols due to the limited availability of pediatric-specific data. Continuous positive airway pressure (CPAP) is typically initiated when upper-airway obstruction from coexisting OSAS predominates, while bilevel PAP may be preferred in cases with persistent hypoventilation despite CPAP. Bilevel positive airway pressure (BiPAP) should be considered first-line in children with severe daytime hypercapnia, respiratory acidosis, or ongoing nocturnal hypoventilation despite CPAP [50,51].

CPAP improves nocturnal airflow, reduces obstructive events, and enhances oxygenation. When daytime hypercapnia persists despite adequate CPAP use, or when hypoventilation is severe, escalation to BiPAP is recommended. BiPAP provides inspiratory pressure support that more effectively augments alveolar ventilation and reduces PaCO_2_ levels. Although adult studies demonstrate clear benefits of BiPAP in OHS, pediatric-specific comparative data between CPAP and BiPAP are limited [4]. BiPAP may be the preferred first-line modality in children with severe hypercapnia, marked respiratory acidosis, or limited ventilatory reserve [62]. Effective PAP therapy improves gas exchange, reduces daytime sleepiness, enhances functional capacity, and may ameliorate cardiac consequences such as left-ventricular hypertrophy in those with pulmonary hypertension [58]. Adherence to PAP therapy represents a major challenge in pediatric populations, particularly among adolescents [58]. Discomfort, mask intolerance, social stigma, and interference with daily routines frequently limit effective use. Even among children meeting conventional adherence thresholds (≥4 h per night), persistent hypercapnia may occur due to insufficient nightly use or suboptimal settings. These challenges underscore the need for regular follow-up, individualized mask fitting, behavioral support, and family engagement to optimize adherence. Supplemental nocturnal oxygen may be required in severe cases but should only be used after ensuring adequate ventilation and is often discontinued as nocturnal respiratory control improves [58].

### 7.2. Weight-Control Strategies

Weight management is a fundamental component of OHS treatment, as it improves respiratory mechanics, reduces ventilatory load, and enhances the effectiveness of positive airway pressure therapy [12]. However, achieving sustained weight reduction is particularly challenging in children and adolescents. Evidence supporting weight-loss interventions in pediatric OHS is indirect and largely extrapolated from general pediatric obesity studies rather than OHS-specific trials.

In children and adolescents, the primary goal of weight management is to improve BMI trajectory and reduce metabolic risk while preserving normal growth and pubertal development, rather than achieving rapid weight loss [12,53]. Successful management requires a family-centered, multidisciplinary approach that integrates nutritional counseling, physical activity promotion, behavioral therapy, and psychosocial support. Psychosocial barriers, including low motivation, emotional distress, socioeconomic constraints, and family dynamics, may limit adherence and long-term success [53,63,64].

Comorbidities such as hypertension, nonalcoholic fatty liver disease, and type 2 diabetes should be routinely screened for and addressed, as they often coexist with pediatric obesity and may worsen respiratory function [53]. Interventions should be tailored to each child’s developmental stage, health status, and psychosocial context to ensure safe and effective outcomes.

### 7.3. Healthy Diet and Nutritional Counseling

Nutritional interventions should focus on sustainable, age-appropriate dietary modifications rather than restrictive regimens. Strict very-low-calorie diets are generally discouraged in pediatric populations due to poor adherence and potential psychological harm [53]. Educational strategies such as structured meal planning, reduction of sugar-sweetened beverages, and increased intake of fruits, vegetables, and whole grains are preferred [58,61]. While these approaches are supported by pediatric obesity guidelines, no studies have specifically evaluated their impact on gas exchange or hypercapnia in pediatric OHS, highlighting a major knowledge gap.

### 7.4. Physical Activity

Regular physical activity contributes to improvements in body composition, cardiovascular fitness, and ventilatory efficiency. Pediatric recommendations suggest at least 20–60 min of moderate-to-vigorous activity daily [65].

Exercise programs should be individualized, considering joint limitations, cardiorespiratory fitness, and patient preferences [53]. Although physical activity is a cornerstone of pediatric obesity treatment, its specific role in reversing hypoventilation in pediatric OHS remains unclear.

### 7.5. Pharmacological Therapy

Pharmacological treatment plays a limited and largely experimental role in pediatric OHS. Weight-loss medications, including orlistat and GLP-1 receptor agonists such as liraglutide and semaglutide, have demonstrated efficacy for improving BMI and metabolic outcomes in adolescents [53,66,67]. However, their effects on pediatric OHS-specific outcomes, such as PaCO_2_ and gas exchange, have not been studied. Therefore, these agents should be considered adjuncts to guideline-directed obesity management, rather than primary therapies for OHS [53,66,67].

Orlistat is currently the only medication approved for pediatric use; its efficacy is modest, and gastrointestinal side effects and reduced absorption of fat-soluble vitamins (A, D, E, K) require careful monitoring [53]. Emerging pharmacologic agents, including GLP-1 receptor agonists, show promise in selected adolescents with obesity, but data remain limited, and their role in pediatric OHS management is not established [66,67].

In children with coexisting sleep-disordered breathing and asthma, leukotriene receptor antagonists such as montelukast may provide additional benefit, particularly in the presence of adenotonsillar hypertrophy. Inhaled corticosteroids remain the preferred controller therapy for children with mild persistent asthma. Importantly, these agents target coexisting conditions and do not directly treat OHS itself [68].

Experimental respiratory stimulants, such as medroxyprogesterone or acetazolamide, have been studied mainly in adults and should be regarded as rescue or adjunctive options in highly selected pediatric patients under specialist supervision [31,61,69,70,71]. Non-invasive ventilation and weight management remain the cornerstone of pediatric OHS treatment.

Therefore, pharmacological therapies should not be considered standard treatment for pediatric OHS but may be regarded as experimental or rescue options in highly selected patients and only under specialist supervision [22].

### 7.6. Bariatric Surgery

Bariatric surgery is the most effective intervention for severe obesity refractory to conservative treatment and may improve ventilation and gas exchange. However, its role in pediatric OHS is constrained by strict eligibility criteria, including pubertal maturity, psychosocial readiness, and long-term adherence capacity. Evidence for bariatric surgery in pediatric OHS is indirect and extrapolated from adolescent obesity studies and adult OHS cohorts. Moreover, remission of OHS is not guaranteed, and residual sleep-disordered breathing frequently persists [72,73]. Premature discontinuation of PAP therapy after surgery, particularly common among adolescents, may compromise outcomes [53]. Therefore, bariatric surgery should be considered only in highly selected adolescents within specialized centers and integrated into long-term multidisciplinary care [74].

### 7.7. Tracheostomy

Tracheostomy is reserved for children with severe, refractory OHS who are unable to tolerate PAP therapy or have persistent nocturnal obstruction despite optimal treatment. By bypassing the upper airway, tracheostomy can correct anatomical obstruction and improve ventilation. Nonetheless, respiratory-effort-related events may persist, and the procedure carries significant implications for quality of life. It should therefore be considered only as a last resort or in highly complex patients [7,53,58].

## 8. Management of Complications

Polycythemia is an uncommon but clinically significant complication of OHS and results from chronic hypoxemia. Initial management involves optimization of non-invasive ventilation to correct hypoventilation and improve gas exchange.

In rare, severe cases refractory to ventilation (as in the case reported by Wesley Branstiter et al. [52] of a 12-year-old child with a hematocrit of 69%), therapeutic phlebotomy may be required to reduce blood viscosity and improve tissue oxygenation. In this case, two sessions removing approximately 10% of blood volume led to a reduction in hematocrit to 56.6% and normalization of gas exchange. Adjunctive therapy, such as low-dose aspirin, may be considered to reduce thrombotic risk in patients with marked polycythemia [52].

## 9. Limitations

This narrative review has several important limitations that should be acknowledged. First, the available pediatric literature on obesity hypoventilation syndrome is extremely limited and heterogeneous. Most published studies consist of small retrospective cohorts, case series, or isolated case reports, with sample sizes typically ranging from single cases to fewer than 10–30 children, and only rarely exceeding 40–50 patients across multicenter retrospective analyses. As a result, the overall number of reported pediatric OHS cases remains small and fragmented, precluding robust aggregation of patient-level data or reliable estimates of disease prevalence and outcomes.

Second, the existing evidence is subject to substantial selection bias, as children described in the literature are often referred to tertiary or quaternary centers and tend to represent more severe or clinically overt forms of OHS, frequently associated with advanced obesity, significant sleep-disordered breathing, or cardiopulmonary complications. Milder or earlier phenotypes of pediatric OHS are likely underrecognized and underreported.

Third, there is a lack of standardized diagnostic criteria for pediatric OHS, including age-specific thresholds for hypercapnia, sleep-related hypoventilation, and ventilatory control impairment. This heterogeneity limits comparability across studies and complicates the extrapolation of adult-derived definitions to children and adolescents.

Fourth, most data are observational and retrospective in nature, with a paucity of prospective or interventional studies evaluating diagnostic strategies, ventilatory support modalities, or long-term outcomes. Consequently, many management recommendations are extrapolated from adult studies or based on expert opinion rather than high-quality pediatric evidence.

Finally, geographic representation is uneven, with most reports originating from high-income countries, which may limit the generalizability of findings to regions with different obesity prevalence, healthcare access, and diagnostic resources.

## 10. Conclusions

Pediatric OHS represents a rare but increasingly recognized complication of severe childhood obesity, arising from the interaction of mechanical respiratory constraints, impaired ventilatory control, sleep-disordered breathing, and neurohormonal dysregulation. Although traditionally described in adults, available evidence indicates that children and adolescents may develop chronic hypercapnia earlier and with fewer compensatory mechanisms, underscoring the importance of timely recognition.

Diagnosis remains challenging due to the absence of validated pediatric-specific criteria and the frequent overlap with obesity-related OSAS. A high index of suspicion is therefore required in severely obese children with sleep-disordered breathing, unexplained daytime symptoms, or biochemical markers suggestive of chronic CO_2_ retention. Early identification is critical to prevent progression to chronic respiratory failure and cardiopulmonary complications.

Management relies on a multimodal approaches centered on non-invasive ventilation and comprehensive weight management, with careful attention to adherence and long-term follow-up. However, current recommendations are largely extrapolated from adult studies and limited pediatric case series.

Future research should prioritize the following: (1) the development of age-specific diagnostic criteria, including normative thresholds for PaCO_2_ and serum bicarbonate; (2) prospective pediatric cohorts, to define the natural history of OHS and the trajectory from OSAS to OHS; and (3) interventional studies evaluating non-invasive ventilation strategies and weight-management or pharmacologic interventions using ventilatory and cardiometabolic outcomes.

Addressing these gaps is essential to improve early diagnosis, guide evidence-based management, and ultimately improve outcomes for children and adolescents with OHS.

## Figures and Tables

**Table 2:** Differences between obstructive sleep apnea syndrome and obesity hypoventilation syndrome. Important note on pediatric criteria: Although OHS is defined in adults by BMI ≥ 30 kg/m^2^, pediatric definitions rely on BMI percentiles, with obesity defined as a BMI ≥ 95th percentile for age and sex. Due to limited pediatric data, adult OHS diagnostic criteria are currently applied to children.

Parameter	OSAS	OHS
Pathophysiology	Recurrent upper-airway obstruction during sleep [2]	Alveolar hypoventilation due to obesity-related mechanical load and impaired ventilatory control [9]
Role of Obesity	Common but not required for diagnosis [2]	Key diagnostic criterion (BMI ≥ 30 kg/m^2^ in adults; ≥95th percentile in children) [6,53]
Daytime PaCO_2_	Typically normal; nocturnal hypercapnia may occur [2,54]	Chronically elevated PaCO_2_ (>45 mmHg) during wakefulness [6]
Nocturnal Breathing Events	Recurrent apneas and hypopneas with intermittent hypoxemia [2]	May coexist with OSAS or occur independently; persistent nocturnal hypoventilation [9]
Primary Ventilatory Support	CPAP [55]	BiPAP (bilevel PAP) [50,51]
Other Therapeutic Options	Weight loss; adenotonsillectomy or other ear, nose, or throat surgery [2]	Weight reduction (essential component of management) [12]
Underlying Ventilatory Drive	Reduced ventilatory drive [56]	Impaired central ventilatory drive [57]

## Data Availability

No new data were created or analyzed in this study.

## Abbreviations

The following abbreviations are used in this manuscript:

OHS	Obesity Hypoventilation Syndrome
CDC	Centers for Disease Control and Prevention
OSAS	obstructive sleep apnea syndrome
BMI	Body Mass Index
PaCO_2_	Partial Pressure of Carbon Dioxide
CPAP	Continuous Positive Airway Pressure
BiPAP	Bilevel Positive Airway Pressure
ERV	Expiratory Reserve Volume
FRC	Functional Residual Capacity
PAP	Positive Airway Pressure
GLP-1	Glucagon-Like Peptide-1
